# Letter to Dr Maria Giulia Bellicini, MD, Brescia, in response to her letter to the editor of our published paper in ESC Heart Failure (online); Worse long-term outcomes in new-onset HFpEF vs HFrEF and HFmrEF: findings from the Stockholm PREFERS study

**DOI:** 10.1093/eschf/xvag140

**Published:** 2026-05-19

**Authors:** Hans Persson, Anton Winderud, Camilla Hage, Carin Corovic Cabrera, Ulrika Löfström, Patrik Lyngå, Taner Arslan, Karin Knudsen Malmqvist, Maria J Eriksson, Bengt Persson, Håkan Wallén, Mattias Ekström, Cecilia Linde

**Affiliations:** Department of Cardiology, Danderyd Hospital, Stockholm 182 88, Sweden; Department of Clinical Sciences, Karolinska Institutet, Danderyd Hospital, Stockholm 182 88, Sweden; Department of Cardiology, Danderyd Hospital, Stockholm 182 88, Sweden; Department of Clinical Sciences, Karolinska Institutet, Danderyd Hospital, Stockholm 182 88, Sweden; Department of Medicine, Karolinska Institutet, Stockholm, Sweden; Department of Cardiology, Karolinska University Hospital, Stockholm, Sweden; Department of Cardiology, South Hospital, Stockholm, Sweden; Department of Clinical Sciences and Education, Södersjukhuset, Karolinska Institutet, Stockholm, Sweden; Department of Medicine, Karolinska Institutet, Stockholm, Sweden; Department of Cardiology, Capio St.Göran Hospital, Stockholm, Sweden; Department of Cardiology, South Hospital, Stockholm, Sweden; Department of Clinical Sciences and Education, Södersjukhuset, Karolinska Institutet, Stockholm, Sweden; Translational Science & Experimental Medicine, Research and Early Development, Cardiovascular, Renal and Metabolism (CVRM), BioPharmaceuticals R&D, AstraZeneca, Gothenburg, Sweden; Department of Cardiology, Danderyd Hospital, Stockholm 182 88, Sweden; Department of Clinical Sciences, Karolinska Institutet, Danderyd Hospital, Stockholm 182 88, Sweden; Department of Clinical Physiology, Karolinska University Hospital, Stockholm, Sweden; Department of Molecular Biology and Medicine and Surgery, Karolinska Institutet, Stockholm, Sweden; Science for Life Laboratory, Department of Cell and Molecular Biology, Uppsala University, Uppsala, Sweden; Department of Medical Biochemistry and Biophysics, Science for Life Laboratory, Karolinska Institutet, Stockholm, Sweden; Department of Cardiology, Danderyd Hospital, Stockholm 182 88, Sweden; Department of Clinical Sciences, Karolinska Institutet, Danderyd Hospital, Stockholm 182 88, Sweden; Department of Cardiology, Danderyd Hospital, Stockholm 182 88, Sweden; Department of Clinical Sciences, Karolinska Institutet, Danderyd Hospital, Stockholm 182 88, Sweden; Department of Medicine, Karolinska Institutet, Stockholm, Sweden; Department of Cardiology, Karolinska University Hospital, Stockholm, Sweden

**Keywords:** HFpEF, HFmrEF, HFrEF, New onset, Outcome, HF clinics

We thank Dr Bellicini for her interest in our paper and her relevant questions and considerations.

Our response will be presented here in order of Dr Bellicini’s questions:

**Q1:**  *‘the definition of HFpEF relied on an E/e’ threshold >8, which falls within the indeterminate range and does not reliably indicate elevated filling pressures (2). This raises concerns regarding the inclusion of patients without clear evidence of haemodynamic congestion.’*

**Response:** We acknowledge that the E/e′ threshold (>8) differs from the later 2021 ESC Guidelines recommendation (>9). Patient inclusion in our study began 2015, prior to publication of the 2021 ESC Guidelines, and the chosen threshold was based on prevailing evidence and clinical practice at that time, including previous studies of heart failure (HF) with preserved ejection fraction (EF) in both AMI and chronic HF populations.

Importantly, the echocardiographic characteristics of our HFpEF cohort indicate that the majority of included patients met contemporary diagnostic criteria. The median E/e′ was 12.5, and even the lower quartile was 9, showing that most patients had values well above the current guideline’s threshold (and comparable to those observed in patients with HFrEF).

Accordingly, HFpEF diagnosis in our study was based on an integrated clinical assessment, incorporating symptoms, signs, and complementary investigations. All patients were evaluated in university hospital–based HF clinics by experienced cardiologists and specialist nurses, ensuring rigorous differentiation of HF from alternative diagnoses. This comprehensive approach reduces the risk of including patients without true haemodynamic congestion.

Additional considerations regarding NT-proBNP are addressed below.

**Q2:**  *‘the NT-proBNP thresholds used, >125 ng/L in outpatients and >300 ng/L in hospitalized patients) are non-specific for rule in HF and may reflect comorbidity burden rather than true heart failure, particularly in an elderly population with a high prevalence of atrial fibrillation and other systemic conditions (3).’*

**Response:** Thank you for this important point. At the time of study planning, available evidence and our prior clinical experience supported the use of an NT-proBNP threshold >125 ng/L to identify possible HF in non-hospitalized patients, particularly in outpatient settings. For hospitalized patients, a higher threshold was applied.

Importantly, NT-proBNP levels in our cohort were substantially above these minimum thresholds. The median NT-proBNP level was nearly three times higher than 300 ng/L, and even the lower quartile exceeded 300 ng/L by approximately 50%, indicating that most patients had clearly elevated values. This reduces the likelihood that NT-proBNP elevation was driven solely by comorbidity burden rather than HF.

As with E/e′, NT-proBNP was not used in isolation but as part of an integrated diagnostic approach. As described above, the diagnosis of HF was based on a comprehensive clinical assessment in specialized settings. We agree that HF is a clinical syndrome and that invasive haemodynamic assessment is not feasible in all patients. In this context, the combined use of clinical evaluation, NT-proBNP, and echocardiographic parameters represents a pragmatic and clinically accepted approach to diagnosing HFpEF.

**Q3:** ‘*Although multivariable adjustment was performed, it remains difficult to disentangle the effect of HFpEF itself from the overall clinical profile of these patients, given the interplay between age, comorbidities, and the HFpEF definition. Even after adjustment, the observed association may therefore reflect the underlying clinical phenotype rather than a distinct disease effect. A more cautious interpretation of HFpEF as a diagnostic category would strengthen the clinical implications of the study.’*

**Response:** We agree that, despite multivariable adjustment, it is challenging to fully disentangle the effect of HFpEF as a diagnostic category from the broader clinical profile of these patients, including age and comorbidity burden. Our findings should therefore be interpreted in the context of HF as a clinical syndrome rather than as evidence of a distinct disease effect attributable solely to HFpEF.

Given the severe clinical outcomes observed over relatively short follow-up periods across the HF spectrum—irrespective of ejection fraction, as shown both in large randomized trials and in our cohort—we believe that a diagnostic approach prioritising sensitivity is clinically appropriate, even at the expense of specificity. This approach reflects current practice, where HF is diagnosed using a combination of echocardiography, clinical assessment, and biomarkers, acknowledging the inherent variability of measures such as ejection fraction.

In this context, our results are informative in demonstrating clear differences in treatment response across HF phenotypes. While we found substantial improvements in both EF and NT-proBNP in HFrEF, and a similarly large decrease in NT-proBNP in HFmrEF, no corresponding improvements were observed in the HFpEF group. These findings support a cautious interpretation of HFpEF as a diagnostic category and highlight the persistent unmet need for effective, evidence-based therapies in this population.

Historically, in HFrEF, ejection fraction has played a central role in clinical diagnosis and in guiding treatments that improve outcome, despite considerable inter-observer and inter-machine variability. Today, evidence from randomized controlled trials supports up to six to eight treatments that reduce mortality and readmissions in HFrEF and likely also in HFmrEF.

We strongly believe that continued research will similarly find effective treatment options also for HFpEF. Our prospective study demonstrates that established diagnostic criteria for HFpEF, including a positive clinical assessment of a HF syndrome, identify a high-risk group of patients with poor outcomes, a population for whom few effective therapies were available until recently. This, in contrast and in comparison, to well-treated patients with HFrEF and HFmrEF, in whom adjusted clinical outcomes over a median follow-up of 3.8 years were more favourable and accompanied by substantial biochemical and echocardiographic improvements in EF and NT-proBNP.

## Declarations

### Data Availability

No data were generated or analysed for this manuscript.

